# Benzyl Isothiocyanate and Resveratrol Synergistically Alleviate Dextran Sulfate Sodium-Induced Colitis in Mice

**DOI:** 10.3390/foods13132078

**Published:** 2024-07-01

**Authors:** Jianan Liu, Qian Zhang, Hongshun Hao, Jingran Bi, Hongman Hou, Gongliang Zhang

**Affiliations:** 1 School of Food Science and Technology, Dalian Polytechnic University, No. 1, Qinggongyuan, Dalian 116034, China; ljn1263778194@163.com (J.L.); bijingran1225@foxmail.com (J.B.); houhongman@dlpu.edu.cn (H.H.); 2 College of Food Science and Engineering, Jilin University, Changchun 130062, China; zhangqian7386@163.com; 3 Department of Inorganic Nonmetallic Materials Engineering, No. 1, Qinggongyuan, Dalian 116034, China; beike1952@163.com

**Keywords:** benzyl isothiocyanate, resveratrol, synergistic effect, gut microbiome, DSS-induced colitis

## Abstract

The aim of our study was to investigate whether the combination of benzyl isothiocyanate (BITC) and resveratrol (RES) has a synergistic effect on the inhibition of inflammation in colitis. The results revealed that the BITC and RES combination (BITC_RES) was more effective than either substance alone at significantly alleviating the symptoms of dextran sodium sulfate (DSS)-induced colitis in mice, including the prevention of colon shortening and loss of body weight, a reduction in the disease activity index, and prevention of colon damage. Similarly, compared with the DSS group, BITC_RES reduced myeloperoxidase (MPO) and inducible nitric oxide synthase (iNOS) levels in the mouse colon by 1.4–3.0-fold and 1.4-fold, respectively. In addition, the combination of BITC and RES upregulated the inflammatory factor IL-10 by 1.3- and 107.4-fold, respectively, compared to the individual BITC and RES groups, whereas the proinflammatory factors, including TNF-α, IL-1β, and IL-6, were downregulated by 1.1–7.4-, 0.7–3.6-, and 0.6–2.6-fold, respectively, in the BITC_RES group compared with the individual groups. Gut microbiome analysis indicated that BITC_RES remodeled the structure of gut bacteria at the phylum, family, and genus levels, upregulating the abundance of the phylum Bacteroidetes and the family *Muribaculaceae* and the genus norank_f_*Muribaculaceae* and downregulating the abundance of the phylum Firmicutes. Significant correlations between the relative levels of these proinflammatory cytokines and changes in the gut microbiota were found using Pearson’s correlation analysis. BITC and RES exhibited synergistic effects by reshaping the gut microbiota and modulating the level of serum cellular inflammatory factors, thus exerting a protective effect against colitis.

## 1. Introduction

The main symptoms of ulcerative colitis (UC) are diarrhea, abdominal pain, and weight loss [1,2,3]. Although UC is prevalent worldwide, its pathogenesis is yet to be thoroughly explored. Several factors, including cytosolic inflammatory factors, the gut microbiome, the gut immune system, and the environment [4,5], have been implicated in the progression of UC. The severity of UC relief is related to the antioxidant capacity of the administered substance [6]. Natural products with good antioxidant properties, including polyphenols [7] and isothiocyanates [8], can relieve UC without side effects. Therefore, the use of natural products for UC mitigation is highly important.

Resveratrol (3,5,4′-trihydroxy-trans-stilbene, RES) is a natural polyphenolic compound with a broad spectrum of bioactive health-promoting properties, including anti-inflammatory, antioxidative, and anti-UC effects [9]. Resveratrol has been shown to suppress UC progression in studies using mouse models [10]. RES can reduce the severity of colon damage, inflammation, and mortality in mice and may affect multiple pathways, including cytokine expression, redox status, and cellular signaling in the gut [11]. Importantly, synergistic effects allow lower concentrations of drugs to be used in combination with each other to achieve the same effect as higher concentrations alone, reducing the concentration of drugs used and thus providing a better balance [12]. Previous studies have reported synergistic antioxidant effects of RES and curcumin and synergistic reductions in hypertension in animal models [13]. *Polygonum cuspidatum Siebold & Zucc* extracts inhibit the NF-kappa B signaling pathway and exert their anti-colitic effects through the synergistic action of polysaccharides, RESs, or rhodopsin [14]. Like other isothiocyanates, benzyl isothiocyanate (BITC) has been shown to have significant pharmacological activities, including anti-inflammatory, antimicrobial [15], and antioxidant activities [16]. In addition, allyl isothiocyanate improves dextran sodium sulfate (DSS)-induced UC in mice by increasing tight junction and mucin expression [8]. It has been reported that phenethyl isothiocyanate treatment attenuates the symptoms of DSS-induced UC in mice [17]. We previously found that BITC and RES synergistically inhibited the growth of *Listeria monocytogenes* by inhibiting biofilm formation, cell membrane disruption and ROS generation [12]. However, no studies have reported synergistic effects of BITC and RES on UC. Therefore, it is necessary to study the synergistic effects of BITC and RES in relieving UC.

The gut microbiota potentially plays a role in the pathogenesis of UC [18]. RES improves enteritis by targeting the colon flora and inflammatory signaling pathways [19]. It also regulates the gut microbiota by inducing Tregs and inhibiting Th17 cells to prevent the development of colitis in mice [20]. By enhancing the ratio of Firmicutes to Bacteroidetes, suppressing the growth of *Enterococcus faecalis*, and improving the abundance of *Lactobacillus* and *Bifidobacterium*, resveratrol has been reported to reduce colon dysbiosis [21]. RES and *Lactococcus lactis* have potential synergistic effects on regulating the colon flora [22]. In addition, sulforaphane (a kind of natural isothiocyanate) alters the microbiota and attenuates colitis severity in DSS-induced UC in mice [23]. ITC-rich moringa seed extract modulates the gut microbiota and thus fat in model mice [24]. Therefore, it is necessary to alleviate DSS-induced colitis by investigating the synergistic modulatory effects of BITC and RES on the colon flora.

Therefore, the aim of this study was to investigate the synergistic mitigating effect of BITC and RES on colitis. First, antioxidant tests were conducted with different ratios of BITC and RES to determine the optimal ratio of synergistic antioxidants. The synergistic effects of BITC and RES on DSS-induced colitis were subsequently assessed by evaluating myeloperoxidase (MPO) and inducible nitric oxide synthase (iNOS) levels; the levels of inflammatory factors, including IL-10, TNF-α, IL-1β and IL-6; and changes in the colon flora.

## 2. Materials and Methods

### 2.1. Materials

BITC (purity > 99%) was acquired from Sigma Aldrich (St. Louis, MI, USA). Resveratrol (RES, 99%) was acquired from Shanghai Aladdin Biochemical Co., Ltd. (Shanghai, China). Anhydrous ethanol was procured from the Damao Chemical Factory (Tianjin, China). All the other chemicals used were of analytical grade.

### 2.2. Antioxidant Activity

#### 2.2.1. DPPH Radical Scavenging Activity Assay

A previously described method was used to determine the DPPH scavenging capacity of the samples [25], with some modifications. BITC and RES were dissolved in ethanol at ratios of 1:1, 1:2, 1:3, 1:4, 1:5, 5:1, 4:1, 3:1, and 2:1. Briefly, 2 mL of 100 µM DPPH ethanol solution was mixed with equal amounts of BITC and RES and then incubated at room temperature in the dark for synergistic antioxidant testing. After 30 min, the absorbance at 517 nm was measured. The DPPH• scavenging percentage was estimated using the following equation:

$$DPPH\;scavenging\;percentage\left(\%\right)=\left(\frac{Ac-As}{Ac}\right)\times 100\%$$

where *Ac* is the absorbance of the control reaction and *As* is the absorbance of the test sample.

#### 2.2.2. ABTS•^+^ Scavenging Activity Assay

The method of Floegel et al. was used for the ABTS•^+^ radical scavenging assay [26], with slight modifications. BITC and RES were dissolved in ethanol at ratios of 1:1, 1:2, 1:3, 1:4, 1:5, 5:1, 4:1, 3:1, and 2:1. In brief, ABTS was dissolved in water to prepare the ABTS stock solution. The ABTS stock solution was reacted with the same volume of potassium persulfate for 12–16 h at room temperature in the dark. BITC and RES were dissolved in absolute ethanol. BITC, RES, and BITC_RES were reacted with the ABTS•^+^ solution in the dark. After 6 min of reaction, the absorbance was measured at 734 nm using a UV-visible spectrophotometer. To calculate the percentage of ABTS•^+^ radicals, the following equation was used:

$${ABTS•}^{+}scavenging\;percentage\left(\%\right)=\left(\frac{Ac-As}{Ac}\right)\times 100\%$$

where *Ac* is the absorbance of the control reaction and *As* is the absorbance of the test sample.

### 2.3. Experimental Animals

Male BALB/c mice (5 weeks) were purchased from Liaoning Changsheng Biological Co., Ltd., Benxi, Liaoning, China. All mice were housed in the SPF standard animal house of the Animal Experiment Center of Dalian University of Technology at a temperature of 25 ± 2 °C, a humidity of 60 ± 5%, and a 24-h period of day and night alternation (license number DLPU2023033). All animals were fed regularly according to the IACUC-approved protocol for laboratory animals prior to the experiments. The animals were fed ad libitum with deionized water and standard dietary materials, and the bedding was changed once every 3–5 days.

### 2.4. DSS-Induced Colitis in Mice

A total of 50 specific pathogen-free male BALB/c mice (5 weeks of age, weight: 20 ± 0.5 g) were randomized into the control, DSS, BITC, RES, and BITC-RES groups (*n* = 6 per group). BITC and RES were administered intragastrically according to the body weights of the mice. According to Ibrahim et al. [27], 30 mg/kg/d RES was administered intragastrically. In our preliminary experiment, BITC and RES had a strong synergistic antioxidant effect at a 1:1 ratio. Therefore, the dose of BITC in the selected group was also 30 mg/kg/d. In the BITC group and the RES group, the mice were given BITC (30 mg/kg/d) and RES (30 mg/kg/d), and in the BITC_RES group, the mice were given BITC-RES (15 mg/kg/d + 15 mg/kg/d) for 14 days. Starting from the eighth day, 3.5% DSS was given ad libitum to all groups except the healthy control group for 7 days to induce colitis. The mice were fasted for 12 h before euthanasia. The serum, intestine and colon contents of all the mice were collected and stored at −80 °C. Some of the intestine was fixed in paraformaldehyde for further analysis.

### 2.5. Assessment of the Disease Activity Index (DAI)

The DAI was collected from the first day of DSS treatment and scored according to weight loss (scored as 0, no change; 1, <5% loss; 2, 5–10% loss; 3, 10–20% loss; and 4, >20% loss), stool consistency (scored as 0, normal; 2, loose stool; and 4, diarrhea), and fecal blood (scored as 0, none; 2, blood; and 4, gross bleeding) [28]. The final macroscopic result for each animal was the sum of these three separate degrees.

### 2.6. Histological Analysis

The colon tissues were dissected and stained with hematoxylin and eosin (H&E) to identify pathological changes in the distal colon tissues of the mice [29]. First, mouse colon and colon tissues were washed in flowing water for 4 h, embedded, and sectioned at 5 μm. The treated tissues were then placed on slides. The slides were dried, deparaffinized, and embedded in H&E. The degree of colon tissue damage in each group was assessed, and the tissues were photographed by a fluorescence microscope (Nikon, Eclipse Ti, Japan).

### 2.7. Inducible Nitric Oxide Synthase (iNOS) Level and Myeloperoxidase (MPO) Activity Assay

Briefly, for iNOS measurements, a 0.1 g/mL tissue homogenate was prepared with saline and centrifuged at 3000 rpm for 10 min, after which the supernatants were obtained. iNOS production was measured using a nitric oxide synthase assay kit (Nanjing Jiancheng Bioengineering Institute, Nanjing, China) according to the manufacturer’s instructions by evaluating the total volume of reaction liquid, sampling volume, colorimetric light path, reaction time, and tissue homogenate protein concentration. A BCA protein test kit (Tiangen Biotech, Beijing, China) was used to measure the protein concentration. Colon tissue was accurately weighed, an MPO test kit (Nanjing Jiancheng Bioengineering Institute, Nanjing, China) reagent was added as a buffer solution, and 0.05 g/mL tissue homogenate was prepared according to a weight: volume ratio of 1:19. The MPO levels were measured with a mouse MPO test kit according to the manufacturer’s protocol.

### 2.8. Determination of the Serum Proinflammatory Cytokine Concentration

After 12 h of fasting, blood samples were taken from the retroorbital sinuses at the end of the study and centrifuged at 2000× *g* for 15 min. Mouse IL-6, IL-1β, and TNF-α level and IL-10 enzyme-associated immunosorbent test (ELISA) kits (Neobioscience Technology, Shenzhen, China) were used to quantify the serum IL-6, IL-1β, TNF-α, and IL-10 concentrations via ELISA.

### 2.9. Microbial Analysis

After collection, the fecal samples were stored at −80 °C. DNA was extracted from the total microbial community according to the instructions of the E.Z.N.A.^®^ Soil DNA Kit (Omega Biotek, Norcross, GA, USA). PCR amplification of the V3–V4 variable region of the 16S rRNA gene was conducted with 338F (ACTCCTACGGGGAGGCAGCAG) and 806R (GGACTACHVGGGGTWTCTAAT) following the following amplification procedure: 27 cycles of pre-denaturation at 95 °C for 3 min. Then, the mixture was stably heated at 72 °C for 10 min and finally stored at 10 °C (PCR instrument: ABI GeneAmp^®^ Model 9700, Applied Biosystems, Foster City, CA, United States). The PCR system included 4 μL of 5× TransStart FastPfu Buffer, 2.5 mM dNTPs, 0.8 μL of upstream primer (5 µM), 0.8 μL of downstream primer (5 µM), 0.4 μL of TransStart FastPfu DNA polymerase, and 10 ng of template DNA, and had a final volume of 20 μL. Library construction was performed using the NEXTFLEX Rapid DNA-Seq Kit (BIOO Scientific, Austin, TX, USA).

### 2.10. Statistical Analysis

Statistical analysis of the data (from at least three separate experiments) was performed via one-way ANOVA with Duncan’s test for comparison of individual means via SPSS software (v19.0, IBM, Inc., Armonk, NY, USA). *p* < 0.05 was considered to indicate statistical significance.

## 3. Results and Discussion

### 3.1. Synergistic Antioxidant Activities of BITC and RES

**Figure 1:**
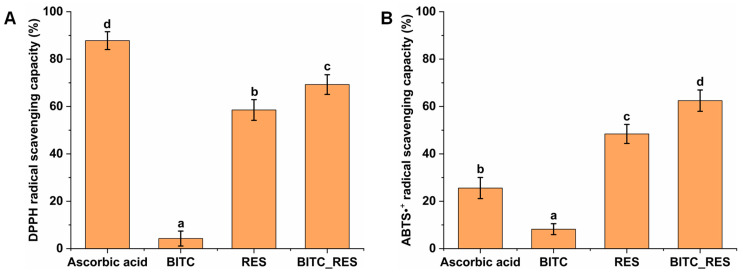
Synergistic antioxidant activities of BITC and RES. DPPH (**A**) and ABTS•^+^ (**B**) radical scavenging capacities. BITC, benzyl isothiocyanate. RES, resveratrol. Different superscript letters in the graph (a, b, c, and d) indicate significant differences (*p* < 0.05).

The antioxidant activities of BITC, RES, and BITC_RES were determined using DPPH and ABTS free radical scavenging activity tests. Finally, the optimal 1:1 ratio of BITC to RES was chosen for the following experiments: BITC (30 μg/mL), RES (30 μg/mL) and BITC_RES (15 μg/mL + 15 μg/mL). DPPH radical scavenging was 69.3% for the mixed BITC and RES samples, which was greater than the 4.3% for the BITC group and 58.5% for the RES group (Figure 1A). However, the level in the ascorbic acid group was significantly greater than that in the BITC_RES group. In addition, the same synergistic effect was observed for ABTS. The ABTS free radical scavenging rate in the BITC_RES group was 1.3-fold to 7.6-fold greater than that in the BITC, ascorbic acid, and RES groups (Figure 1B). These results suggest that BITC and RES have synergistic antioxidant effects. Therefore, we chose an equal concentration ratio to further determine the synergistic effect of BITC and RES on DSS-induced colitis.

### 3.2. Synergistic Effects of BITC and RES on DSS-Induced Colitis Symptom Severity

**Figure 2:**
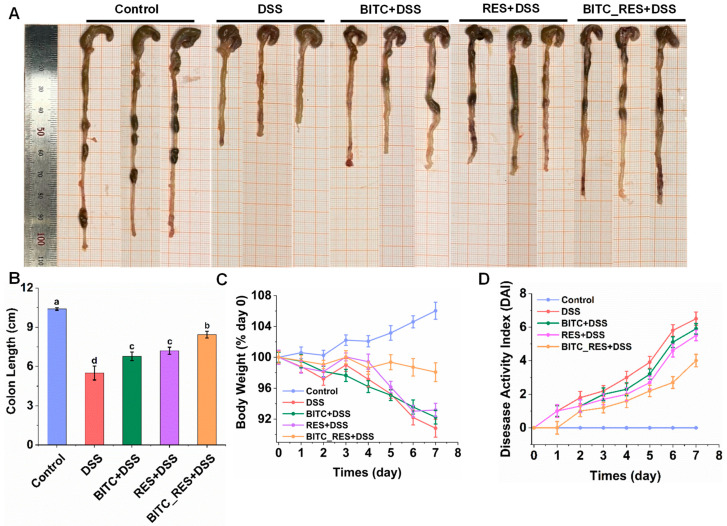
Synergistic effects of BITC and RES on DSS-induced colitis symptom severity. (**A**) Image of the colon length. (**B**) Colon length. (**C**) Percentage of initial body weight. (**D**) Disease activity index score. The data are presented as the mean ± standard error of the mean (*n* = 6). Different superscript letters in the graph (a, b, c, and d) indicate significant differences (*p* < 0.05).

To determine the synergistic effect of BITC and RES on preventing UC, we tested DSS-induced colitis in mice. After DSS treatment, the mice lost weight and developed diarrhea and even hematochezia. The colon length, body weight, and DAI are shown in Figure 2. The control colon tissue was normal in color and thickness, with no congestion, edema or adhesion to surrounding tissue (Figure 2A). The colons of the mice in the DSS group appeared edematous and significantly shorter, with a length of 5.50 ± 0.53 cm (Figure 2A,B). However, mice in the BITC_RES group exhibited particularly significant relief of colonic shortening, with a length of 8.43 ± 0.25 cm, which was 1.17–1.25 times greater than that in the BITC and RES alone groups (*p* < 0.05). In addition, the body weight of the normal mice increased with increasing length of the experimental cycle, which was a natural increase of approximately 106.03% (Figure 2C). The body weights of the mice in the other groups showed different degrees of continuous decline, and the decreasing trend was obvious from the fifth day onward. By the seventh day, the mice in the DSS-induced group exhibited a greater decrease in body weight, down to approximately 90.82% of their starting weight. The BITC and RES groups showed a similar decreasing trend to the DSS group, with body weights decreasing to approximately 92.25% and 93.1%, respectively. However, the weight loss of the BITC_RES-treated mice was significantly reduced, and the body weight decreased to only 98.07%, which was markedly different from that of the other treatment groups. The DAI score is a direct reflection of disease status. As is shown in Figure 2D, the results showed that DAI was dramatically increased in DSS-induced mice to 6.50 ± 0.40 compared to the control group. The BITC and RES groups reduced the DAI scores, and, in particular, the BITC_RES group significantly reduced the DAI score by 2.5 relative to the DSS group (*p* < 0.05). These findings are consistent with previous research [8,10,30,31], demonstrating that ITC and RES prevent the progression of colitis. Therefore, the combined effect of BITC and RES was greater than that of either alone, further suggesting a synergistic effect of BITC and RES.

### 3.3. Synergistic Effects of BITC and RES on Colonic Microinjury in DSS-Induced Colitis

**Figure 3:**
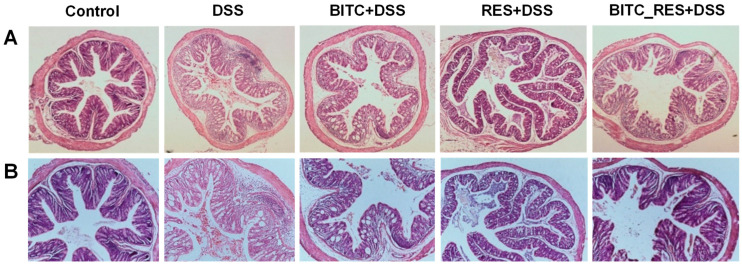
Synergistic effects of BITC and RES on colonic microinjury in DSS-induced colitis. Hematoxylin and eosin staining of representative inflamed areas of colon sections ((**A**), magnification ×40; (**B**), magnification ×100).

We observed changes in the microstructure of the colon in five groups of mice to further study the effect of BITC in combination with RES on colitis (Figure 3). In the control group, the colonic mucosa was intact and continuous, and the glands were regularly arranged and structured. In the DSS group, the colonic tissues were severely lesioned, the epithelial cells of the colonic tissues had detached, the crypts and the structure of the cups had basically disappeared and were disorganized, the submucosal layer was more severely affected, and some areas were congested and severely ulcerated. Compared with that in the model group, inflammatory cell infiltration gradually decreased in the BITC and RES groups. However, the colonic mucosal tissue of the BITC_RES group was more similar to that of the control group, and BITC_RES had a greater preventive effect on UC than did BITC or RES alone. It has been reported that the combination of an anthocyanin-rich extract of black rice and rosemarinic acid inhibits DSS-induced colitis-induced colon damage to varying degrees compared to treatment alone [32]. Therefore, these results suggest that BITC and RES have synergistic effects on DSS-induced colitis-related colonic injury.

### 3.4. Synergistic Effects of BITC and RES on iNOS and MPO Levels in Colonic Tissues and Cytokine Level in Serum

**Figure 4:**
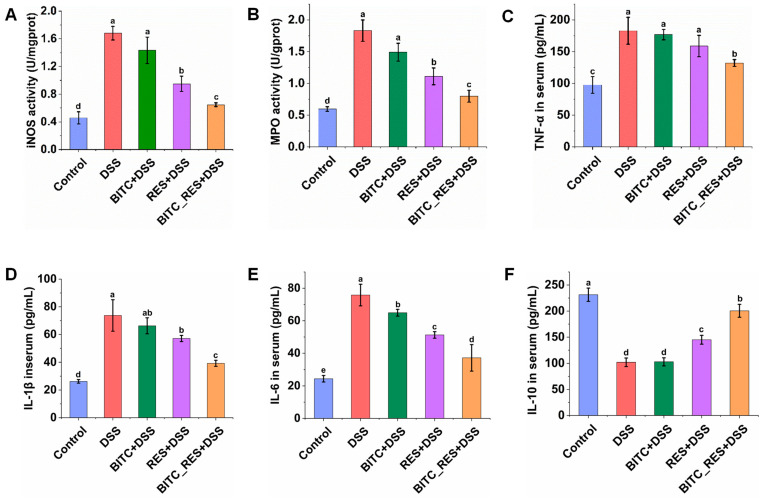
Synergistic effects of BITC and RES on iNOS and MPO levels in colonic tissues and cytokine levels in serum. (**A**) iNOS, (**B**) MPO, (**C**) TNF-α, (**D**) IL-1β, (**E**) IL-6, and (**F**) IL-10. The data are expressed as the mean ± SD (*n* = 6). Bars with different superscripts letters are significantly different.

Excess NO produced by iNOS through direct cytotoxicity, neutrophil activation, vasodilation, and peroxynitrite radical formation could worsen the clinicopathological features of colitis [33]. As shown in Figure 4A, the iNOS level in the control group was 0.46 ± 0.09 U/mg protein, while in the DSS group, it was markedly increased to 1.68 ± 0.10 U/mg protein (*p* < 0.05). Among all the intervention groups, iNOS levels were significantly lower in the BITC_RES and RES groups than in the DSS group, and the former reduced iNOS levels by 1.4 times more than did the latter. There was no significant difference in the BITC group. In addition, compared with that in the control group, the level of MPO in the DSS group decreased from 1.83 ± 0.17 U/g protein to 0.60 ± 0.04 U/g protein. The RES and BITC_RES treatments attenuated this response, and the MPO level in the BITC_RES group was similar to that in the control group (Figure 4B). Similarly, BITC_RES had synergistic effects on the MPO level, with MPO values of 1.49, 1.11, and 0.80 (Figure 4B). Our findings are consistent with previous studies that have shown natural products to be more effective in combination with each other than alone for preventing colitis and lowering NO and MPO levels [32,34]. In addition, TNF-α, IL-1β, and IL-6 are typical proinflammatory cytokines; IL-10 is a classical anti-inflammatory cytokine; and these cytokines are closely linked with the formation and progression of UC [35,36]. Therefore, a potential way to alleviate UC is to restore the balance of these cytokines. The serum cytokine levels were measured to further assess the extent of inflammation (Figure 4C–F). The serum levels of TNF-α, IL-1β, and IL-6 in the control group ranged from 24.4–97.6 pg/mL. Compared with those in the control group, the serum levels of TNF-α, IL-1β, and IL-6 were significantly elevated to 73.7–182.8 pg/mL in the DSS model mice. There was no significant difference in the TNF-α level between the BITC-only group and the RES-alone group compared to that in the DSS group (Figure 4C), but the TNF-α level in the BITC_RES group was significantly lower (50.64 pg/mL). Similarly, BITC_RES cotreatment reduced the level of IL-1β and IL-6 more significantly than BITC or RES alone, as shown in Figure 4D,E (*p* < 0.05). A trend in the opposite direction was observed for the level of the anti-inflammatory cytokine IL-10 (Figure 4F). Although the significant attenuation of colonic tissues’ TNF-α, IL-1β, and IL-6 levels with RES in the DSS group was consistent with the findings of earlier studies [10,37], the modifying effects of BITC on serum TNF-α, IL-1β, and IL-6 have not been previously studied. BITC metabolites revealed that benzylamine was the major metabolite and there were lesser amounts of benzoic acid, benzaldehyde, *N*,*N*′-di-benzylurea, and *N*,*N*′-di-benzylthiourea [38]. Benzylamine substituents play an important role in antioxidant and anti-inflammatory properties [39]. Therefore, the anti-inflammatory effects of BITC may be related to its metabolites. Previous studies have shown that the polysaccharides resveratrol and rhodopsin have synergistic effects on the level of inflammatory factors, which in turn alleviate colitis [14]. Therefore, BITC and RES synergistically regulate the level of inflammatory factors.

### 3.5. Synergistic Effects of BITC and RES on the Colon Microbiota of Mice with DSS-Induced Colitis

**Figure 5:**
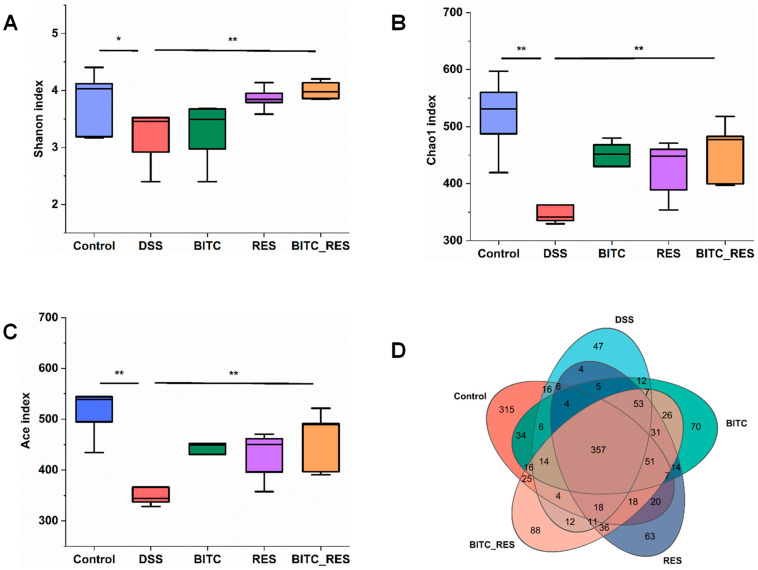
Diversity of the gut microbiota among the control, DSS, BITC, RES, and BITC_RES groups (*n* = 5). α-Diversity analysis of the (**A**) Shannon, (**B**) Chao1, and (**C**) Ace indices. (**D**) Venn diagram of shared and unique species at the OTU level. * *p* < 0.05, ** *p* < 0.01.

The gut microbiome is a complex community of microbes that has an essential impact on the health of the host. UC is strongly linked with the onset and progression of dysbiosis [40]. In this study, we investigated whether BITC and RES have synergistic regulatory effects on the colon flora to ameliorate DSS-induced colonic inflammation in mice. Previous studies have shown that BITC and RES can ameliorate disease by regulating the composition of the gut flora [24,30]. The number of operational taxonomic units (OTUs), Shannon diversity, Chao1 index and Ace index were assessed because they are widely used measures of alpha diversity, indicating the depth of sequence coverage and community diversity within assays [41]. As shown in Figure 5A–C, the α diversity, which included the Shannon, Chao1 and Ace indices, was significantly lower in the DSS group than in the control group. In addition, 357 OTUs were identified as being shared between the different groups, as shown in the Venn diagram. The numbers of specific OTUs in the control group, DSS group, BITC group, RES group, and BITC-RES group were 315, 47, 70, 63, and 88, respectively (Figure 5D). These findings indicate that species diversity was reduced by DSS treatment, while BITC-RES treatment restored species richness more significantly than did BITC or RES treatment alone.

**Figure 6:**
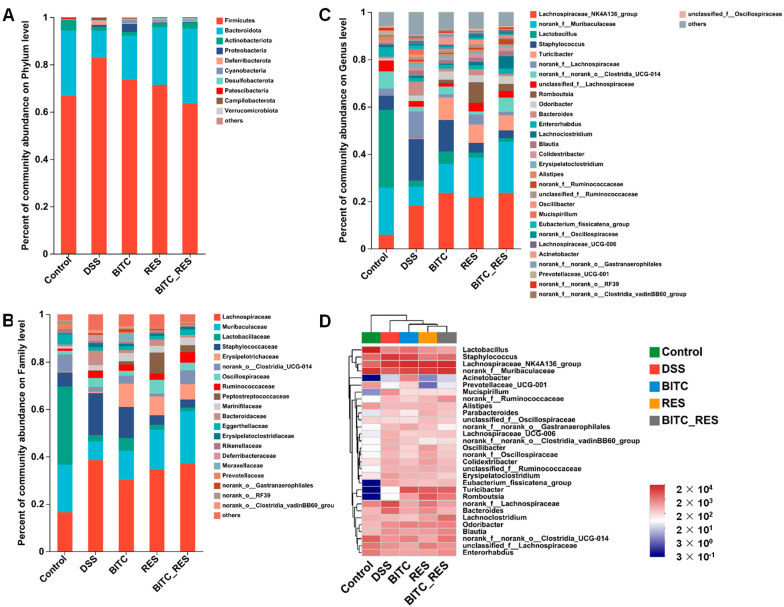
The percent community abundance histogram of the gut microbiota in the control, DSS, BITC, RES, and BITC_RES groups. (**A**) Phylum level, (**B**) family level, and (**C**) genus level. A cluster heatmap of species richness is shown (**D**).

To understand which taxa BITC and RES may influence, we assessed the relative abundances of the dominant taxa within and between treatment groups. As shown in Figure 6A, Firmicutes, Bacteroidetes, Actinobacteria, Proteobacteria, and Deferribacterota were identified at the phylum level in five different groups, with Firmicutes and Bacteroidetes being the two most abundant bacterial phyla. The pathogenesis and regulation of enteritis are closely related to the structure of the colon microbiota [42]. For example, the ratio of Firmicutes to Bacteroidetes is associated with the degree of inflammation in patients with colitis [43]. In the DSS-group, there was an increase in the relative abundance of Firmicutes and a decrease in the relative abundance of Bacteroidetes; a similar conclusion was reached by Li et al. [44]. In contrast, BITC-RES was effective at reversing the Firmicutes-to-Bacteroidetes ratio, as assessed by BITC and RES treatments alone, which was consistent with the findings of previous studies [45]. Bacteroidetes are the largest gram-negative phylum in the microbiome of the gastrointestinal tract and are typically beneficial for hosts when confined to the gastrointestinal tract [46]. Earlier studies have shown that the average level of Bacteroidetes in the UC group was significantly lower than that in the control group [47]. RES also increased Bacteroidetes and decreased Firmicutes in DSS-induced colitis mice and alleviated symptoms, in line with our studies [48]. At the family level, there was a significant enrichment of the beneficial bacteria *Muribaculaceae*, *Lactobacillaceae*, and *Lachnospiraceae* in the control group (Figure 6B). Treatment with DSS resulted in a significant decrease in these OTUs and an increase in the relative abundance of harmful bacteria belonging to Staphylococcaceae and Bacteroidaceae. After RES and BITC_RES treatment, the abundance of beneficial bacteria increased, and the abundance of pathogenic bacteria decreased. Once we had identified the roles of BITC and RES in regulating colon microbial structure at both the phylum and family levels, we sought to identify which bacterium was likely responsible for their effect on chronic colitis in mice. In addition, at the genus level, community abundance histograms and heatmaps were generated to examine the compositions of the communities in the different groups (Figure 6C,D). *Muribaculaceae* are considered anti-inflammatory bacteria for use in evaluating the effectiveness of Lactobacillus plantarum Q7 for preventing ulcerative colitis [49]. *Muribaculaceae* was strongly reduced in the DSS model group compared with the control group and increased in abundance in the BITC and RES treatment groups compared with the BITC- and RES-alone treatment groups. Liu et al. showed that *Muribaculaceae* outperformed the other bacteria in the consumption of inulin, resistant starch and polysaccharides and in the production of propionate [50,51]. According to Wang et al., *Muribaculaceae* improved the levels of short-chain fatty acids when given undigested pea protein [52]. BITC_RES administration resulted in the upregulation of *Staphylococcus* and *Turicibacter* and the downregulation of *Bacteroides* iparison to those in the model group. In conclusion, BITC_RES treatment enhanced the diversity of beneficial bacteria and reduced the diversity of harmful bacteria relative to those in the BITC and RES groups, further demonstrating the synergistic effect of BITC and RES in ameliorating colitis-related inflammation.

**Figure 7:**
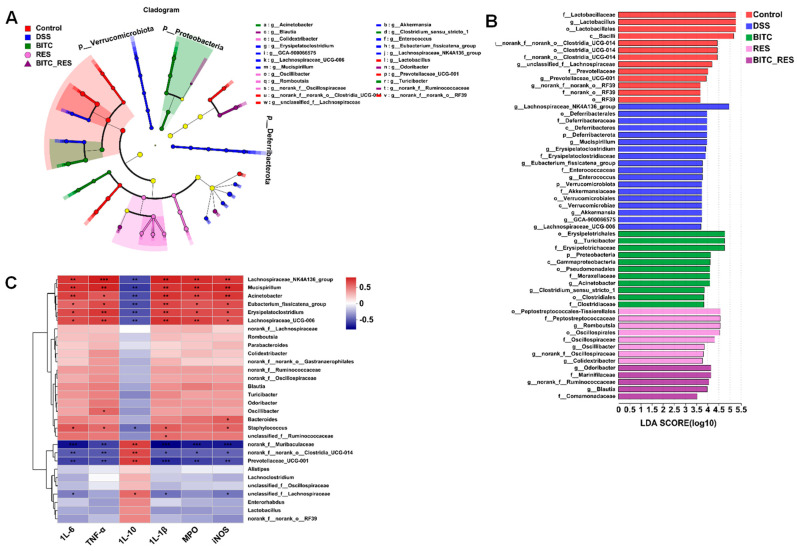
Microbial taxa discrepancies among the control, DSS, BITC, RES, and BITC_RES groups. Linear discriminant analysis (LDA) revealed scores > 3.5 and adjusted *p* values < 0.05. (**A**) Cladogram and histogram (**B**,**C**) correlation analysis (*p* < 0.05 and |R^2^| > 0.7, * *p* < 0.05, ** *p* < 0.01, *** *p* < 0.001) based on Spearman’s rank order correlation between the 30 most abundant gut microorganisms at the genus level and biochemical criterion.

The abundance of the gut microbiota at the phylum and genus levels was also assessed by LEfSe (Figure 7A,B). *Odoribacter* was the major genus in BITC_RES. *Odoribacter* belongs to the order *Bacteroidales* and is a wide short-chain fatty acid-producing constituent of the human gut microbiota [53]. Phenotypic analysis has shown that members of *Bacteroidales* possess a very broad spectrum of sugar degradation capabilities, with some strains capable of degrading tens of different complex carbohydrates [54]. To determine possible associations between the gut microbial composition and pro- and anti-inflammatory cytokines, we examined Spearman’s correlation coefficients among the 30 most abundant gut microbiomes at the genus level and six indicators of colon inflammation (Figure 7C). IL-10 was positively related to the abundance of norank_f_*Muribaculaceae*, norank_f__norank_o__*Clostridia*_UCG-014, *Prevotellaceae*_UCG-001, and unclassified_f__*Lachnospiraceae* and negatively related to the diversity of *Bacteroides* and *Mucispirillum*. Conversely, the levels of inflammatory cytokines (IL-6, IL-1β, and TNF-α) and inflammatory enzymes (iNOS and MPO) correlated positively with the diversity of *Bacteroides* and *Mucispirillum* and negatively with *Lachnospiraceae*_NK4A136_group, *Lachnospiraceae*_UCG-006, and others. The pathogenic genera *Bacteroides* and *Mucispirillum* were also found to induce inflammation in mice by Sheng et al. and Herp et al. [55,56]. Furthermore, Fei et al. reported that combined treatment modified the abundance and metabolism of microorganisms in the gut, specifically affecting the levels of short-chain fatty acids and anti-inflammatory-associated molecules [57]. In conclusion, BITC_RES better reshaped the colon’s bacterial community, altered the levels of inflammatory factors in mice, and consequently improved colonic inflammatory symptoms.

## 4. Conclusions

BITC and RES were found to have synergistic antioxidant effects. BITC_RES increased colon length, alleviated weight loss, decreased DAI values, increased levels of the proinflammatory factor IL-10, and decreased levels of several proinflammatory cytokines, including TNF-α, IL-1β, and IL-6, compared with the BITC and RES groups. In addition, BITC-RES was effective at reversing the Firmicutes-to-Bacteroidetes ratio, as assessed by the BITC and RES groups alone. Therefore, the synergies of BITC and RES can be applied to UC. However, due to the high volatility of BITC and the low bioavailability of RES, it is necessary to study the codelivery system of both.

## Data Availability

The original contributions presented in the study are included in the article, further inquiries can be directed to the corresponding author.

## Abbreviations

BITC	benzyl isothiocyanate
RES	resveratrol
BITC_RES	benzyl isothiocyanate and resveratrol
MPO	myeloperoxidase
DSS	dextran sodium sulfate
NOS	nitric oxide synthase
DAI	disease activity index
OUT	operational taxonomic unit
